# Coital incontinence: a factor for deteriorated health-related quality of life and sexual function in women with urodynamic stress urinary incontinence

**DOI:** 10.1007/s00192-016-3185-3

**Published:** 2016-11-07

**Authors:** Magdalena Emilia Grzybowska, Dariusz Grzegorz Wydra

**Affiliations:** Department of Gynecology, Gynecologic Oncology and Gynecologic Endocrinology, Medical University of Gdańsk, Kliniczna 1a, 80-402 Gdańsk, Poland

**Keywords:** Coital incontinence, Female sexual function, Pelvic organ prolapse, Pelvic Organ Prolapse/Urinary Incontinence Sexual Questionnaire (PISQ), Quality of life, Stress urinary incontinence

## Abstract

**Introduction and hypothesis:**

To assess the impact of coital incontinence (CI) on health-related quality of life (HRQoL) and quality of sexual function (QSF) in women with urodynamic stress urinary incontinence (SUI).

**Methods:**

Women were recruited for this cross-sectional study from among 289 patients with lower urinary tract symptoms, underwent clinical and urodynamic evaluation. Of these 289 women, 127 sexually active women with SUI completed the King’s Health Questionnaire (KHQ) and the Pelvic Organ Prolapse/Urinary Incontinence Sexual Questionnaire (PISQ), of whom 97 were enrolled for the study. The study group comprised 53 women with CI occurring ‘sometimes’, ‘usually’ or ‘always’, and the control group comprised 44 women without CI. Total and individual domain scores were evaluated.

**Results:**

CI was reported by 65.35 % of the women. The frequency of CI was correlated with lower educational level and higher body mass index (*r* = 0.22 and *r* = 0.23, respectively; *p* = 0.01). The KHQ results showed significantly lower HRQoL in women with CI in all domains (*p* < 0.05) apart from Sleep/energy’ (*p* = 0.054). PISQ revealed no significant differences in QSF in the Behavioral/emotive and Partner–related domains (34.3 ± 10.0 vs. 33.0 ± 12.2 and 18.0 ± 2.9 vs. 18.2 ± 3.6, respectively). Women with CI reported a significantly lower QSF in the Physical domain (29.1 ± 6.6 vs. 35.0 ± 4.6, *p* = 0.001), and the total PISQ score was lower but the difference was not significant (81.4 ± 14.3 vs. 86.2 ± 16.5). Total PISQ score was correlated with age (*r* = −0.28, *p* = 0.001). Women with CI were significantly more likely to admit that fear of incontinence or fear of embarrassment restricted their sexual activity (*p* < 0.001).

**Conclusions:**

A large percentage (65.35 %) of women with SUI reported CI, which had a negative impact on HRQoL and QSF in the Physical domain, but no significant impact on overall QSF.

## Introduction

Stress urinary incontinence (SUI) is the most common type of urinary incontinence, affecting up to 40 % of all women [1]. The negative impact of urinary incontinence on quality of life (QoL) and sexual function has been well documented. Sexual dysfunctions have been confirmed in 46 % of women with urinary incontinence and lower urinary tract symptoms (LUTS), and include hypoactive sexual desire (34 %), sexual arousal disorders (23 %), orgasmic dysfunction (11 %), and dyspareunia (44 %) [2]. Approximately 20 % of patients with urinary incontinence and pelvic organ prolapse (POP) discontinue any sexual activity because of these symptoms [3]. According to Lonnée-Hoffmann et al. [4], urinary coital incontinence (CI) affects up to 40 % of women with SUI and 21 % of women with POP. Sexual function in women with SUI has often been investigated, especially before and after surgery, but few studies have focused on the issue of CI. It remains a neglected subject among health-care professionals and constitutes a mere fraction of the numerous questions in general questionnaires assessing QoL in women with urinary incontinence. The symptom is difficult to diagnose because women rarely refer it spontaneously (3 %, in contrast to 20 % on direct questioning) [5]. The effect of CI on the quality of sexual function is not obvious and the data in the literature are conflicting. In a study in The Netherlands, symptomatic improvement following surgical treatment for SUI was shown to correlate with improved sexual function in women with CI compared with women without CI [6]. On the other hand, over half of the affected women perceived the impact of CI on their sexual function as ‘small’ (59.8 %), and 32.3 % perceived it as ‘moderate’ and 7.9 % as ‘considerable’ [7].

In this study we evaluated the impact of CI on QoL and sexual function in women with urodynamically confirmed SUI.

## Materials and methods

Women were recruited for this cross-sectional study from among 289 consecutive patients who attended a urogynecology ambulatory clinic with LUTS. All tests were performed according to the standards recommended by the International Continence Society. Medical history, including a detailed sexual history and CI, was taken. All the women were requested to keep a 48-h voiding diary, and underwent urogynecological examination, assessment of POP using the Pelvic Organ Prolapse Quantification (POP-Q) system, and urodynamic examination. A negative urine culture was mandatory for urodynamic testing, which was performed using a Solar system (Medical Measurement Systems, Enschede, The Netherlands). The inclusion criteria were self-reported sexual activity, literacy and SUI. Women with mixed and urge urinary incontinence, and those who refused consent, were excluded. Overall, 127 sexually active heterosexual women with SUI were recruited.

All participants were asked by a physician to complete the King’s Health Questionnaire (KHQ) and the Pelvic Organ Prolapse/Urinary Incontinence Sexual Questionnaire (PISQ), and were classified according to their reported CI frequency (‘always’, ‘usually’, ‘sometimes’, ‘seldom’ or ‘never’). For the purposes of this analysis, women reporting CI ‘sometimes’, ‘usually’ or ‘always’ were assigned to the study group, and women reporting CI ‘seldom’ were excluded. Women with SUI and without CI comprised the control group. The final comparison groups were 44 women continent during sexual activity (control group) and 53 women reporting CI more often than ‘seldom’ (study group). All patients were stratified according to the Stamey incontinence severity score: *grade 1* urine leakage on strong physical effort (coughing, sneezing), *grade 2* urine leakage on medium effort (change of position), and *grade 3* urine leakage on minimal effort (lying position) [8].

The KHQ has proved to be reliable and valid for evaluation of QoL in patients with urinary incontinence. It includes 21 questions divided into the following eight domains including: General health, Incontinence impact, Role limitations, Physical limitations, Social limitations, Personal relationships, Emotions, and Sleep/energy. The scores for each domain range from 0 to 100, with 0 representing the best and 100 representing the worst possible health status [9]. The Polish version of the KHQ (information, contact and consent) is available from MAPI Research Trust, Lyon, France (e-mail PROinformation@mapi-trust.org; www.mapi-trust.org). The PISQ is used to assess the quality of sexual function in women with POP and urinary incontinence, and the Polish version has been validated [10]. It consists of 31 questions divided into three domains: Behavioral/emotive, Physical, and Partner-related. Low PISQ scores indicate lower quality of sexual function [11].

The study was approved by the local Ethics Committee. All patients gave their informed consent to participate in the study, and their privacy was maintained. The study was conducted in accordance with the principles of the Declaration of Helsinki.

### Statistical analysis

Statistical analysis was performed with SPSS for Windows 17.0 (SPSS, Inc., Chicago, IL). All continuous variables are expressed as means and standard deviations. Categorical variables are expressed as percentages of the total group. A *p* value of <0.05 was considered statistically significant and all statistical tests were two-sided. For the independent tests, Student’s *t* test and the Mann–Whitney *U* test were used. The Mann–Whitney *U* test was used to compare two groups as a nonparametric alternative to Student’s *t*-test (when the variables were ordinal), and the Kruskal-Wallis test was used to compare more than two groups. Statistically significant results were analysed using post-hoc tests. Pearson’s chi-squared test of independence was used to examine the interdependences between categorical data. Spearman’s rho was used to analyse the relationships between variables. Analysis of covariance (ANCOVA) was performed to eliminate the covariates effect.

Altman’s nomogram was used to determine the sample size. Standardized differences were determined based on minimal important differences (MID; a difference that is clinically meaningful to patients) with standard deviations (SD) obtained from the literature [12, 13]. Altman’s nomogram provided values for the assumed probability value of *p* = 0.05. The following results were obtained form the tests: KHQ: N = 60 (approximately 30 subjects in each group), SD = 8.2, MID = 5; PISQ: N = 90 (approximetely 45 subjects in each group), SD = 10.9, MID = 6.

The main outcome measures were KHQ and PISQ scores in women with SUI with or without CI. The questionnaire scores and the scores for the individual domains were assessed. The scores were also correlated with patient body mass index (BMI), age, level of education, degree of SUI, and POP severity in relation to the frequency of CI.

## Results

Of the 289 women with LUTS, 194 with SUI were sexually active and met the inclusion criteria. A total of 127 women (response rate 65.5 %) completed all the questionnaires. The medical history revealed that 44 women (34.6 %) were continent during sexual activity (the control group). Of the remaining women, 27 (21.3 %) who reported CI ‘sometimes’, 18 (14.2 %) ‘usually’ and 8 (6.3 %) ‘always’ comprised the study group, but 30 (23.6 %) who reported CI ‘seldom’ were excluded from the study. The characteristics of the study and control groups are presented in Table 1. The groups did not differ in terms of age, parity, menopause or marital status. The women with CI had a significantly higher BMI than the controls (*p* < 0.03). The frequency of CI was correlated with higher BMI (*r* = 0.23, *p* = 0.01). Women without CI had a higher level of education than women with CI, and the correlation between higher level of education and lower frequency of CI was significant (*r* = 0.22, *p* = 0.01).

**Table 1:** Characteristics of the study population

	Study group (*n* = 53)	Control group (*n* = 44)	*p* value
Age (years), mean ± SD	52.6 ± 8.0	53.6 ± 0.3	>0.05^a^
BMI (kg/m^2^), mean ± SD	28.4 ± 5.5	26.1 ± 4.2	<0.03^a^
Age at menarche (years), mean ± SD	13.6 ± 1.3	13.6 ± 1.6	>0.05^a^
Age at menopause (years), mean ± SD	48.7 ± 5.5	48.2 ± 6.1	>0.05^a^
Postmenopausal, *n* (%)	30 (56.6)	28 (63.6)	>0.05^b^
Parity, mean ± SD (median)	2.5 ± 1.1 (2)	2.3 ± 0.8 (2)	>0.05^c^
Education, *n* (%)			
Primary	11 (20.8)	2 (4.6)	<0.03^b^
Secondary	30 (56.6)	25 (56.8)	
Tertiary	12 (22.6)	17 (38.6)	
Marital status, *n* (%)			
Married	47 (88.7)	36 (81.8)	>0.05^b^
Partnership	6 (11.3)	8 (18.2)	
POP-Q stage, *n* (%)			
0	2 (3.8)	4 (9.1)	>0.05^b^
I	9 (16.9)	4 (9.1)	
II	40 (75.5)	32 (72.7)	
III	2 (3.8)	4 (9.1)	
IV	–	–	
Stamey SUI severity score, *n* (%)			
1	14 (26.4)	25 (56.8)	<0.001^b^
2	24 (45.3)	18 (40.9)	
3	15 (28.3)	1 (2.3)	

a Student’s *t* test

b Chi-squared test

c Mann–Whitney *U* test

There were no significant differences between the women with CI and the controls in terms of a history of anterior repair or vaginal hysterectomy (18.9 % and 9.1 %, respectively; *p* > 0.05), previous abdominal hysterectomy (13.2 % and 9.1 %, respectively), and no history of previous surgery (69.8 % and 75 %, respectively).

### Coital incontinence evaluated with the King’s Health Questionnaire

KHQ scores were compared between women reporting CI and the controls. The results for both groups are presented in Table 2. Women with CI had a significantly lower QoL than the controls in all domains except Sleep/energy, although even in this domain the differences approached borderline statistical significance.

**Table 2:** Mean scores for the KHQ domains in the study and control groups

Domain	Study group (*n* = 53)	Control group (*n* = 44)	*p* value	
			Crude, for Student’s *t* test	Adjusted
General health	53.5 ± 18.9	44.2 ± 23.7	0.04	0.09
Incontinence impact	80.7 ± 22.4	60.5 ± 32.7	0.001	0.002
Role limitations	68.0 ± 26.5	45.7 ± 36.7	0.001	0.002
Physical limitations	71.7 ± 25.0	50.0 ± 33.5	0.001	0.001
Social limitations	40.4 ± 31.2	21.4 ± 26.7	0.002	0.005
Personal relationships	41.5 ± 32.4	16.7 ± 23.6	<0.001	0.001
Emotions	59.3 ± 28.7	42.4 ± 33.0	0.009	0.065
Sleep/energy	45.0 ± 33.9	31.8 ± 30.8	0.054	0.126
Severity measures	73.6 ± 26.2	51.8 ± 32.8	0.001	0.005

The data are presented as means ± SD

The Personal relationships domain of the KHQ included questions pertaining to personal and intimate life, such as: ‘Does your bladder problem affect your relationship with your partner?’, ‘Does your bladder problem affect your sex life?’, and ‘Does your bladder problem affect your family life?’. CI had a substantial influence on the answers selected. Only one in four women with CI denied any effect of their bladder problem in any of the domains, whereas 65.9 % of the controls answered ‘not at all’ to all of these questions. Regarding the question ‘Do you get embarrassed because of your bladder problem?’ from the Emotions domain, 112 (88.2 %) of the sexually active women with SUI were embarrassed by their bladder problem, including 48 (37.8 %) who reported ‘extreme’ embarrassment. All 53 patients (100 %) with CI reported embarrassment due to their bladder problem.

The KHQ results were adjusted for BMI and anal incontinence. ANCOVA revealed that women without CI achieved lower scores (higher QoL) after adjustment for covariables in the following domains: Incontinence impact, Role limitations, Physical limitations, Social limitations, Personal relationships and Severity measures, compared with women with CI. Only the General health perception, the Emotions and the Sleep/energy domains did not differ significantly between the groups. In the KHQ only the Role limitations domain score was correlated with age (*r* = 0.21, *p* = 0.02). The older the subject, the greater was the impact of the bladder problem on household tasks and normal daily activities outside the home.

### Sexual function in relation to coital incontinence evaluated with PISQ

The PISQ Physical domain score was significantly lower in women with CI (*p* = 0.001), and the PISQ total score revealed reduced quality of sexual function, but the difference between the groups was not significant (*p* > 0.05). The results are presented in Table 3. Analysis of covariance confirmed these results. Differences in the reported frequency of CI on the basis of medical history and PISQ (question 18: ‘Are you incontinent of urine with your sexual activity?’) were not significant (*p* > 0.05).

**Table 3:** Mean PISQ total scores and domain scores in the study and control groups

Domain	Study group (*n* = 53)	Control group (*n* = 44)	*p* value for Student’s *t* test adjusted
Behavioral/emotive	34.3 ± 10.0	33.0 ± 12.2	>0.05
Physical	29.1 ± 6.6	35.0 ± 4.6	0.001
Partner-related	18.0 ± 2.9	18.2 ± 3.6	>0.05
Total score	81.4 ± 14.3	86.2 ± 16.5	>0.05

The data are presented as means ± SD

Dyspareunia (‘always’, ‘usually’ or ‘sometimes’) was reported by 12 women (27.3 %) in the control group and by 16 women (30.2 %) with CI (*p* > 0.05). Additionally, 8 women (15.1 %) with CI reported being ‘usually’, ‘sometimes’ or ‘seldom’ incontinent of stool with sexual activity, whereas only one woman in the control group reported ‘seldom’ stool incontinence during sexual activity (*p* = 0.03). Women with CI were significantly more likely to admit that the fear of incontinence or fear of embarrassment restricted their sexual activity (*p* < 0.001). The data from the Physical domain questions are presented in Table 4.

**Table 4:** Responses to the Physical domain questions (PISQ) in the study and control groups

Question	Response	Study group (*n* = 53)	Control group (*n* = 44)	*p* value (Mann–Whitney *U* test)
11. Do you feel pain during sexual intercourse?	Always	1 (1.9)	1 (2.3)	0.6
	Usually	4 (7.6)	2 (4.5)	
	Sometimes	11 (20.7)	9 (20.4)	
	Seldom	18 (34.0)	14 (31.8)	
	Never	19 (35.8)	18 (40.9)	
13. Is your vaginal opening so ‘tight’ that sexual intercourse cannot occur?	Extremely tight	0	0	0.9
	Pretty tight	0	3 (6.8)	
	Somewhat tight	6 (11.3)	5 (11.4)	
	Not very tight	6 (11.3)	1 (2.3)	
	Not tight at all	41 (77.4)	35 (79.5)	
16. Do you avoid sexual intercourse because of bulging in the vagina (either the bladder, rectum, or vagina falling out)?	Always	1 (1.9)	2 (4.5)	0.01
	Usually	4 (7.6)	0	
	Sometimes	12 (22.6)	2 (4.5)	
	Seldom	5 (9.4)	4 (9.1)	
	Never	31 (58.5)	36 (81.8)	
17. Do you engage in anal or oral sex because vaginal sexual activity is uncomfortable for any reason?	Always	1 (1.9)	0	0.35
	Usually	0	0	
	Sometimes	9 (17.0)	2 (4.5)	
	Seldom	6 (11.3)	9 (20.4)	
	Never	37 (69.8)	33 (75.0)	
18. Are you incontinent of urine with sexual activity?	Always	5 (9.4)	0	<0.001
	Usually	13 (24.5)	2 (4.5)	
	Sometimes	21 (39.6)	4 (9.1)	
	Seldom	12 (22.6)	6 (13.6)	
	Never	2 (3.8)	32 (72.7)	
19. Are you incontinent of stool with sexual activity?	Always	0	0	0.03
	Usually	1 (1.9)	0	
	Sometimes	3 (5.7)	0	
	Seldom	4 (7.5)	1 (2.3)	
	Never	45 (84.9)	43 (97.7)	
20. Does fear of incontinence (either stool or urine) restrict your sexual activity?	Always	1 (1.9)	0	<0.001
	Usually	9 (17.0)	0	
	Sometimes	19 (35.8)	3 (6.8)	
	Seldom	5 (9.4)	7 (15.9)	
	Never	19 (35.8)	34 (77.3)	
21. Does fear of embarrassment due to incontinence restrict your sexual activity?	Always	6 (11.3)	2 (4.5)	<0.001
	Usually	10 (18.9)	0	
	Sometimes	16 (30.2)	3 (6.8)	
	Seldom	10 (18.9)	7 (15.9)	
	Never	11 (20.7)	32 (72.7)	
25. When you have sex with your partner, do you have negative emotional reactions such as fear, disgust, shame or guilt?	Always	3 (5.7)	1 (2.3)	0.03
	Usually	6 (11.3)	1 (2.3)	
	Sometimes	12 (22.6)	7 (15.9)	
	Seldom	10 (18.9)	9 (20.4)	
	Never	22 (41.5)	26 (59.1)	
30. Do you avoid sexual intercourse because of embarrassment?	Always	4 (7.5)	0	0.001
	Usually	5 (9.4)	1 (2.3)	
	Sometimes	11 (20.7)	2 (4.5)	
	Seldom	8 (15.1)	8 (18.2)	
	Never	25 (47.2)	33 (75.0)	

The data are presented as number (%)

Coital leakage on vaginal penetration was reported by 30 women (36.1 %) with CI, coital leakage on orgasm by 15 (18.1 %), and on both by 38 (45.8 %). Of the 127 sexually active women with SUI analysed in the study, 14 (11 %) had finally abstained from sexual activity, including 7 (5.5 %) who reported urinary incontinence as the main cause.

According to the medical histories, 71.4 % of women with grade 3 SUI experienced CI ‘sometimes’, ‘usually’ or ‘always’ compared with 29.8 % of women with grade 1 SUI and 40.7 % with grade 2 SUI. Post-hoc analysis confirmed that women with grade 3 SUI more often reported CI than women with grade 1 or 2 SUI (*p* = 0.001 and *p* = 0.052, respectively). Urinary incontinence during sexual activity did not occur in 53.2 % of women with grade 1 SUI, in 30.5 % with grade 2 SUI, and in only 4.8 % with grade 3 SUI (Table 5). Although CI was more frequently observed in women with more severe grades of SUI, there was no significant correlation between those two parameters.

**Table 5:** Frequency of coital incontinence in relation to stress urinary incontinence grade

Coital incontinence	No. of women	SUI grade		
		1	2	3
Never	44	25 (53.2 %)	18 (30.5 %)	1 (4.8 %)
Seldom	30	8 (17.0 %)	17 (28.8 %)	5 (23.8 %)
Sometimes, usually, always	53	14 (29.8 %)	24 (40.7 %)	15 (71.4 %)
Total	127	47	59	21
*p* value^a^		<0.001		

^a^Level of significance for ANOVA rank Kruskal-Wallis test, independent variable: frequency of CI

No significant differences in the frequency of CI in relation to POP stage were found, nor was there a significant correlation. In the study group, CI with varying frquency was reported by 65.2% of women with POP-Q stage 0 or I and by 65.4 % of women with POP-Q stage ≥II (*p* > 0.05). The correlations between age and PISQ scores were as follows: Behavioral/emotive domain (*r* = −0.32, *p* < 0.001), Physical domain (*r* = −0.01, *p* > 0.05), Partner-related domain (*r* = −0.32, *p* < 0.001), and total score (*r* = −0.28, *p* = 0.001). Age was correlated with a lower quality of sexual function in the Behavioral/emotive and Partner-related domains and with overall sexual function as assessed by the total PISQ score.

## Discussion

Women with urinary incontinence often experience a fear of uncontrolled leakage of urine during sexual intercourse, and are more likely to report dyspareunia and vaginal dryness, resulting in decreased libido and lower self-esteem [14]. Urinary incontinence during sexual intercourse affects 10.6 – 45 % of women with urinary incontinence and is most common in women with SUI (66.2 %) [7, 15–17]. In our study, 65.35 % of women with SUI suffered from CI. The percentage was lower (41.7 %) after excluding women who reported CI ‘seldom’, which demonstrates the importance of precision regarding questions about frequency. The analysis may be more accurate if the exact number of urinary incontinence episodes is assessed, or the percentage of CI episodes is expressed in relation to the number of sexual intercourses [18]. Interestingly, CI has been confirmed in 14 % of continent women without pelvic floor disorders [14].

Hilton [16] suggested dividing CI into ‘incontinence on penetration’ and ‘incontinence during orgasm’. However, this does not take into account the physical exertion that accompanies sexual activity. Correlations between CI on penetration and SUI, and between CI during orgasm and detrusor overactivity have been found [15, 19]. Urine leakage during penetration is explained by the lowering of the bladder fundus and the urethra during an increase in the intraabdominal pressure and a change in the position of the bladder neck during penetration, whereas an orgasm may cause involuntary detrusor contraction and relaxation of the urethra [20].

The prevalence of CI was also found in 2.2 % women with an overactive bladder, and in 17.5 % with mixed urinary incontinence [15]. Our study group included only patients with SUI, which did not allow comparisons between CI incidence in women with different types of urinary incontinence. An association between SUI severity and CI incidence was observed: women with severe SUI more frequently reported CI. No differences were found in the occurrence of CI during penetration and orgasm.

Urinary incontinence itself has a significant adverse effect on women’s QoL, while the combination of urinary incontinence and CI results in an even poorer QoL [1, 7, 9, 17, 21]. The results of this study are consistent with reports of significantly lower health-related QoL in women with SUI and CI compared with women with SUI without CI. CI significantly affects various aspects of life, including personal relationships, social aspects, role limita﻿ti﻿ons, and physical ability, as well as sleep and energy.

Sexual function assessed with PISQ was affected adversely in women with CI compared with controls, but the differences were significant only in the Physical domain. POP is more often regarded as a factor associated with sexual dysfunction than urinary incontinence itself [3, 22]. Ozel et al. [22] found that in women with urinary incontinence those with POP-Q stage ≥II were more likely than those with POP-Q stage 0 or I to report absence of libido (53 % vs. 30 %, *p* = 0.02), lack of sexual arousal during sexual intercourse (46 % vs. 27 %, *p* = 0.05), and rarely-experienced orgasm during sexual intercourse (49 % vs. 30 %, *p* = 0.05). No relationship between POP stage and CI frequency was found in our study. Moran et al. [15] found that the incidence of CI was higher among women who had undergone anterior repair with or without vaginal hysterectomy; a similar finding was also observed in our patients.

Although patients perceive POP as the basic factor adversely affecting sexual activity, overall sexual satisfaction remains largely unchanged between before and after surgery [3]. This further shows that age and psychological factors such as self-perceived body image have a decisive impact on the quality of sexual function [3, 18, 23, 24]. Sexual function in women with urinary incontinence and POP has also been reported to be comparable with that in healthy women, and that the percentage of sexually active women is similar in both groups [18, 23].

A high BMI is a risk factor for urinary incontinence and is a significant predictor of sexual inactivity [23, 25], although other studies have failed to show a link between obesity and lower quality of sexual function in women with urinary incontinence [26, 27]. Obesity is associated with chronic elevation of abdominal pressure, and the greater pressure exerted on the pelvic floor during sexual intercourse may explain the CI in obese women. In our study, women with CI had a considerably higher BMI than the controls. Madhu et al. [28] confirmed that obesity is a significant risk factor for CI in women with LUTS and CI.

Interestingly, in this study women with CI were less well educated than women who were continent during sexual activity. The literature offers no comparisons of educational level in similar groups of patients. This finding may be explained by the fact that women with better education, considering these symptoms unacceptable, use preventive strategies more effectively. The mechanisms of coping with CI include urinating prior to sexual intercourse, deferring intercourse, interrupting intercourse prematurely, avoiding certain positions, hurrying through sex, and avoiding orgasm [21, 24]. A large impact on QoL in women with urinary incontinence has been shown to be associated with lower educational level [29].

The multiphase contact with patients is a definite strength of our study. This allowed us to obtain a detailed medical history, a sexual history, a precise urodynamic diagnosis, and to perform a physical examination to assess POP. A complex diagnostic process that resulted in a good doctor–patient rapport is the probable reason for the high percentage of women who reported CI in this study. However, our study was limited by the relatively small sample size and the fact that all participants were actively seeking medical help, or were referred to our urogynecological centre. Over 74 % of the group had POP-Q stage II and therefore the comparisons between patients with different POP-Q stages were limited. Nonetheless, we were able to compare health-related QoL and sexual function in a highly selected group of patients.

## Conclusions

CI is a common symptom in patients with urodynamically confirmed SUI. In our study, the prevalence was higher in patients with a high BMI and in those with a low educational level. Health-related QoL assessed with the KHQ and the quality of sexual function assessed with the PISQ Physical domain were both significantly lower in women with SUI and CI than in women with SUI without CI.
